# Emmet’s Operation

**Published:** 1879-06

**Authors:** Robert Barnes

**Affiliations:** London, 15 Harley Street, W.


### Article XV.

London, 15 Harley Street, W., May 3, 1879.

*To the Editor of the Chicago Medical Journal and Examiner :*

Sir : My attention has been called to an article in your issue of March last, on "Emmet's Operation," by Dr. E. C. Dudley, in which occurs the following passage: "Either from absolute "ignorance, or from profound prejudice, or from a carelessness "which in the law would amount to malice, the most exhaustive "gynæcological works in England (Barnes), France (Leblond), "and Germany (Hegar, Kaltenbach, Schrœder) are absolutely "silent."

"Big words!" It is not my affair to inquire how far they may be justly applied to Leblond, Hegar, Kaltenbach and Schrœder. As to me they possibly might be applied, *if the allegation were only true* that I had been "absolutely silent" about "Emmet's operation." But as a matter of fact I have mentioned it, and with the unqualified respect which I entertain for one of the most able and illustrious of my American friends. At page 873, 2d edition, of my "Diseases of Women," 1878, I give a compendious summary of Emmet's operation, quoting the source (*American Journal of Obstetrics*, 1874), and I conclude with the following appreciation: "I can confirm the accuracy of Emmet's views. I have performed his operation with satisfactory results."

So, if there is "absolute ignorance" it is not mine; if there is "profound prejudice," it is not mine; and if there is "carelessness amounting to malice," it is not mine. It is possible that Dr. Dudley is misled by copying a similar impeachment made against me by Dr. Paul Mundé, in the *American Journal of Obstetrics*, instead of reading my book for himself? Dr. Mundé has since apologized to me for his error. Dr. Dudley will no doubt do the same. As I am entitled to damages I claim the right to fix the penalty. I condemn him to read my book, and caution him never again to accept statements at second hand. I am, sir, yours sincerely, Robert Barnes.

